# Management of cognitive symptoms in schizophrenia.

**DOI:** 10.1192/j.eurpsy.2024.1567

**Published:** 2024-08-27

**Authors:** T. Jupe, I. Kourtesis, I. Sarris

**Affiliations:** ^1^ Psychiatric Hospital of Attica; ^2^Thriasio General Hospital of Elefsina, Athens, Greece

## Abstract

**Introduction:**

Cognitive impairment is the frequent symptom occurring in nonelderly patients with schizophrenia or other neurodegenerative disorders. Cognitive dysfunction in patients with schizophrenia was described by Kraepelin more than a century ago. Increased awareness and advancements in the area of neuropsychological assessment and neuroimaging techniques have now rendered cognitive impairment an important focus of theories on the etiology and treatment of schizophrenia. Cognitive enhancement still remains a clinically unresolved challenge. Till date, there is no effective treatment available for enhancing cognitive function in patients with schizophrenia

**Objectives:**

This e-poster aimed to summarize evidence regarding clinical data on the nonpharmacologic and pharmacologic management of cognitive symptoms of schizophrenia, also known as cognitive impairment associated with schizophrenia (CIAS), and highlight the selection of appropriate treatment options.

**Methods:**

A bibliopgraphical review was performed using PubMed platform. All relevant articles were found using the keywords: schizophrenia, cognitive symptoms, menagement.

**Results:**

Many different drug targets and strategies for drug development have been employed for enhancement of cognition in schizophrenia. Receptor targets have been identified on the basis of pharmacologic challenges that mimic schizophrenia (e.g., dopamine agonists and NMDA receptor antagonists), receptor abnormalities found on postmortem analysis of schizophrenia brain, and genetic linkage studies. Treatment with a D_1_ agonist was shown to “sensitize” D_1_ receptors and improve memory in both aged and antipsychotic-treated monkeys. Clinical trials of D_1_ agonists in schizophrenia have been delayed due to poor tolerability related to orthostatic hypotension and nausea. The glycine-site agonists, glycine, D-serine, and D-alanine, produced improvement in negative symptoms in small trials and some improvement in measures of cognition, although formal cognitive testing was not performed.

**Conclusions:**

Although the evidence supporting cognitive remediation has not yet achieved the level necessary to merit inclusion in evidence-based treatment guidelines, this approach, combined with other psychosocial interventions, is promising. Several pharmacologic approaches are currently under study to facilitate neuroplasticity.

**Disclosure of Interest:**

None Declared

